# Prevalence of palliative sedation in the State of São Paulo: an emerging medical demand

**DOI:** 10.31744/einstein_journal/2020AO5395

**Published:** 2020-09-09

**Authors:** Márjorie Anção Oliveira Piedade, Carlos Alberto Cardoso, Denise Gonçalves Priolli

**Affiliations:** 1 Universidade São Francisco Bragança PaulistaSP Brazil Universidade São Francisco, Bragança Paulista, SP, Brazil.; 2 Escola de Educação Física e Esporte Universidade de São Paulo São PauloSP Brazil Escola de Educação Física e Esporte, Universidade de São Paulo, São Paulo, SP, Brazil.

**Keywords:** Deep sedation, Palliative care, Pain management, Bioethics, Terminal care, Advance care planning

## Abstract

**Objective:**

To investigate the prevalence of palliative sedation use and related factors.

**Methods:**

An observational study based on data collected via electronic questionnaire comprising 23 close-ended questions and sent to physicians living and working in the state of São Paulo. Demographic data, prevalence and frequency of palliative sedation use, participant’s familiarity with the practice and related motivating factors were analyzed. In order to minimize memory bias, questions addressing use frequency and motivating factors were limited to the last year prior to survey completion date. Descriptive statistics were used to summarize data.

**Results:**

In total, 20,168 e-mails were sent and 324 valid answers obtained, resulting in 2% adherence. The overall prevalence of palliative sedation use over the course of professional practice was 68%. However, only 48% of respondents reported having used palliative sedation during the last year, primarily to relieve pain (35%). The frequency of use ranged from one to six times (66%) during the study period and the main reason for not using was the lack of eligible patients (64%). Approximately 83% of physicians felt comfortable using palliative sedation but only 26% reported having specific academic training in this field.

**Conclusion:**

The prevalence of palliative sedation use is high, the primary indication being pain relief. However, frequency of use is low due to lack of eligible patients.

## INTRODUCTION

Maintaining the quality of life of patients with advanced disease and refractory symptoms^(1)^ is a major challenge for healthcare professionals.^(2,3)^ These scenarios call for emerging strategies such as palliative sedation, a palliative care approach consisting of deliberate administration of drugs to reduce the level of conscience in these individuals, for proper relief of one or more refractory symptoms.^(4,5)^

Palliative sedation is indicated primarily for hyperactive delirium, dyspnea, severe intractable pain, uncontrollable bleeding and myoclonia,^(6,7)^ with consent given by patient or guardian/person responsible. According to data reported in literature, the prevalence of palliative sedation use among healthcare professionals ranges from 1% to 88%.^(8,9)^ Such wide range may be justified by different palliative sedation definitions adopted in different studies; guidelines and culture of countries in which palliative sedation is practiced;^(10-12)^ understanding of refractory symptoms; and experience and comfort of health professionals;^(9)^ place of palliative sedation application; prevalence assessment methods; and type of public interviewed.^(8)^

Palliative sedation is widely used to treat physical symptoms.^(6,7)^ However, treatment of psycho-existential symptoms remains debatable,^(8,13)^ given the difficulty in detecting appropriate responses to disease and mental disorders, such as depression. Also, the technique remains a challenging therapeutic dilemma, due to the potential misunderstanding of continuous deep sedation and euthanasia.^(8,14)^ Continuous deep palliative sedation leads to consciousness suppression until death, and is intended to alleviate suffering. In contrast, euthanasia is defined as the act of accelerating death of a patient by means of lethal doses of drugs and is intended to deliberately end life to interrupt suffering.^(14,15)^

In Brazil, discussions involving this technique are recent,^(16)^ and specific guidelines addressing palliative sedation indications are lacking. In 2009, palliative care was included as a fundamental principle in the Code of Medical Ethics by the Federal Medical Council and, in 2011, Palliative Medicine was officially recognized as a medical specialty. A resolution addressing palliative care organization within the National Health System of Brazil (SUS - *Sistema Único de Saúde*) has been recently published.^(17)^ However, significant impacts of this ordinance in medical practice have yet to be reported.

Likewise, research addressing palliative care and sedation in the hands of Brazilian professionals are scarce. The prevalence of palliative sedation is thought to range from 17% to 37%,^(18,19)^ dyspnea, delirium and pain being the major reasons for indication.^(19)^ Family-related issues stand out as a major hurdle to palliative sedation implementation.^(19)^ Nonetheless, these data were extracted from studies with small sample size and focusing on patients or multidisciplinary teams in specialty center settings. Specific data regarding medical practice of palliative sedation is therefore lacking.

## OBJECTIVE

To investigate the prevalence and frequency of palliative sedation use is the State of São Paulo, Brazil. Major motivations and hurdles to implementation of the technique and whether physicians living and working in São Paulo are trained in palliative care were also investigated.

## METHODS

### Experimental design

This article was written according to Strengthening the Reporting of Observational Studies in Epidemiology (STROBE) recommendations.^(20)^ An observational, cross-sectional epidemiological study based on data collected using an electronic questionnaire adapted from two previous studies,^(9,19)^ and implemented via Google Forms. The questionnaire comprising 23 objective questions was distributed to a community of physicians from the State of São Paulo, between March and November 2017 (Annex 1).

### Participants

Physicians living and working in the state of São Paulo, with at least one year of experience in clinical practice (sole inclusion criterion). Volunteers failing to complete the survey or providing duplicate responses were excluded. Prior to starting the survey (first page of the form), participants were informed of study details and filled out an Informed Consent Form.

### Procedures

Participants were recruited via the Regional Medical Council of the State of São Paulo (CREMESP) registration. Three e-mail contact attempts, 15 days apart, were made to each of the 20,168 e-mail addresses obtained. Contact attempts to 32 medical societies, 2 associations and 2 academies requesting them to forward the questionnaire link to their members, or to provide access to the survey on their official websites, were also made.

### Questionnaire

Participants answered a questionnaire comprising 23 objective questions online. Demographic data (sex, age, time in medical practice and specialty) were interrogated first. Palliative care and palliative sedation concepts were then introduced^(4,5,21)^ and participants interrogated as to whether they were in favor of palliative sedation and considered the technique to be equivalent to euthanasia or end-of-life practice; whether palliative sedation was a well-established practice in their workplace; whether physical symptoms might impact psycho-existential symptoms; and whether they would indicate palliative sedation to treat non-physical symptoms.

The prevalence of palliative sedation use was also investigated. In order to minimize memory bias, some questions were limited to the 12 months prior to questionnaire completion,^(22)^ such as prevalence of use during this time frame, major drivers, frequency of palliative sedation use, level of comfort regarding drug administration, palliative care-specific training and impact of family members on decision making regarding the use of palliative sedation. Physicians who had never used palliative sedation or had not used it over the last twelve months prior to questionnaire completion, were inquired about major reasons for not doing so.

### Statistical analysis

Findings were summarized using descriptive statistics and frequency analysis. Statistical analyses were conducted using IBM^®^ SPSS^®^ Statistics version 23.

This study was conducted in compliance with Ethical Standards and Guidelines, with the approval of the Research Ethics Committee of *Universidade São Francisco* (number 1.964.009, CAAE: 65240217.4.0000.5514).

## RESULTS

A total of 338 questionnaires were obtained out of 20,168 e-mails sent and contacts made with aforementioned institutions. Fourteen questionnaires were excluded due to duplicity (12) or incomplete fields (2). The final sample comprised 324 questionnaires selected for analysis.

Demographic data of volunteers are shown in table 1. The sample comprised 51% of male participants (n=165). Prevailing age ranged from 30 to 39 years (34%) and 44% of participants reported more than 16 years of professional experience. A total of 314 interviewees were specialist physicians (97%), and most were practicing internal medicine (n=167).

**Table 1:** Socio-professional profile of physicians with more than one year of experience

Sex	
Male	165 (51)
Female	159 (49)
Age, years	
<30	55 (17)
30-39	110 (34)
40-49	6 (20)
50-59	47 (19)
60-69	42 (13)
>70	7 (1)
Professional experience, years	
1	19 (6)
2-5	7 (22)
6-10	4 (15)
11-16	45 (14)
>16	141 (44)
Specialist	
Yes	314 (97)
No	10 (3)
Specialties	
Internal Medicine	167 (53)
Surgery	60 (19)
Pediatrics	26 (8)
Psychiatry	17 (5)
Ginecology and Obstetrics	16 (5)
Family Medicine	10 (3)
Occupational Medicine	8 (3)
Anesthesiology	6 (2)
Others	4 (1)

Results expressed as n (%).

Most interviewees (99%) were in favor of palliative sedation practice and did not believe palliative sedation to be equivalent to euthanasia (312; 99%) or end-of-life practice (278; 83%). However, around 60% (n=195) believed palliative sedation not to be properly established at their place of work. All physicians believed physical symptoms to impact psycho-existential symptoms; still, 61% (n=198) of them would not indicate palliative sedation to patients with non-physical symptoms.

**Figure 1:**
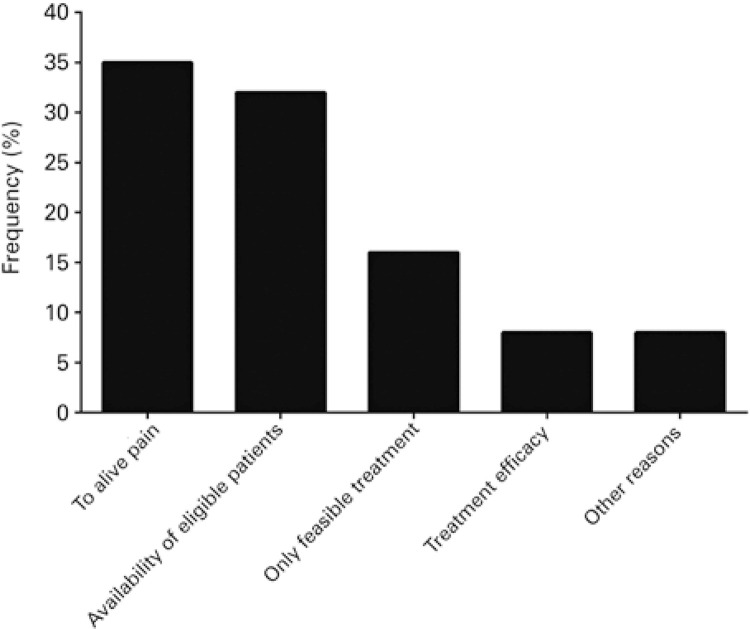
Distribution of physicians with 1 or more years of experience according to reasons for palliative sedation practice

**Figure 2:**
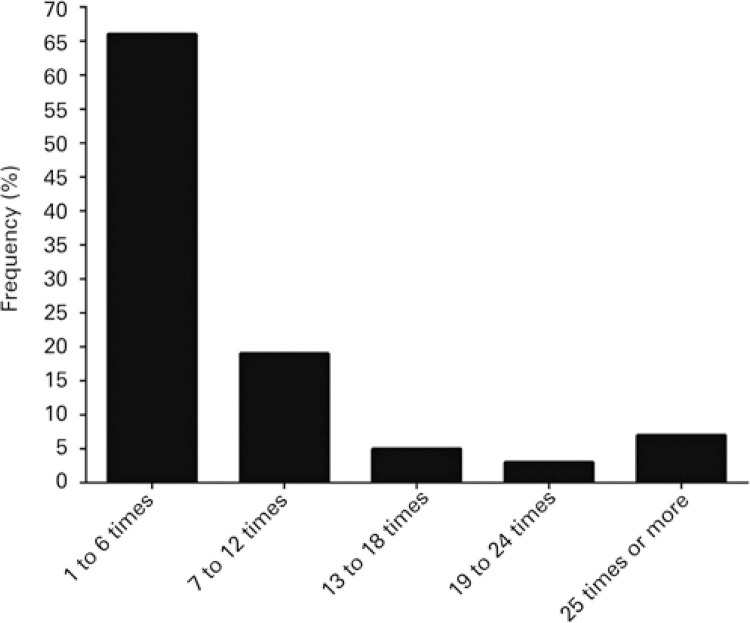
Distribution of physicians with 1 or more years of experience according to frequency of palliative sedation use

**Figure 3:**
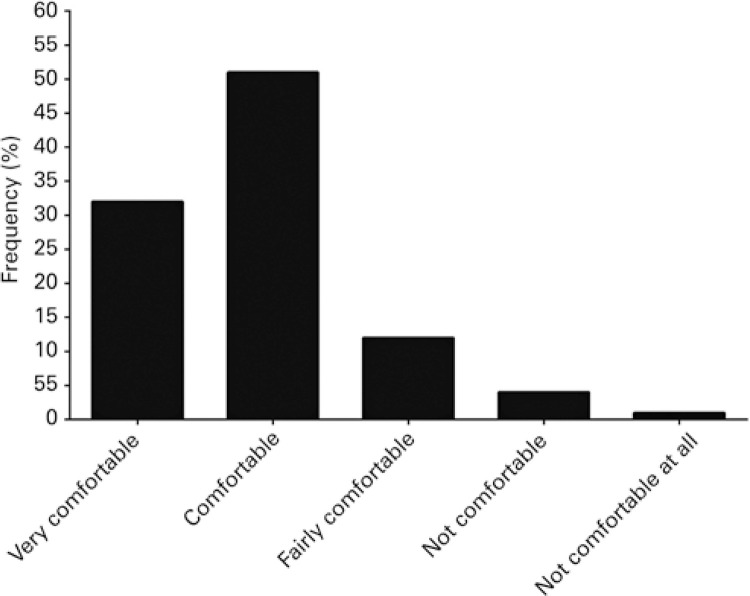
Distribution of physicians with 1 or more years of experience according to level of comfort about drug selection for palliative sedation

When inquired about the use of palliative sedation, 68% (n=221) reported having employed the technique as a treatment alternative for eligible patients. The prevalence of palliative sedation use over the course of the last 12 months prior to questionnaire completion was 48% (n=155). Palliative sedation was indicated primarily to alleviate suffering (n=97; 35%) (Figure 1) and 66% (n=103) of interviewees reported having used palliative sedation between one and six times during the year (Figure 2). Around 83% (n=129) of professionals reporting use of palliative sedation over the last 12 months felt comfortable or very comfortable about drug selection (Figure 3). However, only 26% (n=41) of professionals reporting use of palliative sedation declared having specific training in palliative care. Also, 95% (n=148) of physicians reported consulting or having consulted family members prior to the procedure, and 86% (n= 133) believed patient families may play a role in establishing the practice at healthcare settings.

The major reason given for not using palliative sedation among those who had never used it or had not used it over the last 12 months was the lack of eligible patients (64% and 100%; n=66 and n=66, respectively). Not being aware of the procedure (n=35; 34%) and other, non-specified reasons (n=2; 2%) were other justifications given for never having used the technique.

## DISCUSSION

High prevalence of palliative sedation use among physicians working in São Paulo was the major finding of this study. However, prevalence decreased when the analysis was limited to the last twelve months and frequency of use was relatively low. Only a minority of physicians had specific training in palliative care and the major reason for palliative sedation use was complaint of pain.

This sample comprised mostly male physicians (51%), physicians with more than 16 years of professional experience and physicians aged between 30 and 39 years. Also, 97% of participants are specialists. These findings are in keeping with data reported by CREMESP, in 2018: 55% of males among the 126,687 professionally active physicians working in the state of São Paulo, mean age of 45 years, 20.2 years of professional experience and 66% were specialists.

In spite of the subtle boundaries between palliative sedation and euthanasia reported in the literature and potential misunderstanding of both practices,^(14,15)^ findings of this study suggest healthcare professionals participating in this survey were able to differentiate between them, a factor that may legitimate the use of palliative sedation in eligible patients. Similar findings have been reported elsewhere:^(15,19)^ health professionals working in an oncologic palliative care hospital sector^(15)^ and those working or having worked in specialized palliative care centers^(19)^ were able to clearly distinguish between both terms, and referred cited ethics as the foundation supporting the practice of palliative sedation.

Interviewees in this sample believed physical symptoms to be associated with emotional symptoms. Still, findings of this study indicated most professionals would not use palliative sedation to treat patients suffering from psycho-existential pain. Increased indication of palliative sedation for treatment of psycho-existential symptoms on bioethical grounds has been reported.^(23)^ However, its use for treatment of non-physical symptoms has also been contraindicated, since these are not always associated with imminent death.^(24)^

The prevalence of palliative sedation in this study was 68% overall and 48% when the analysis was limited to twelve months prior to data collection. These findings reflect literature data suggesting the prevalence of palliative sedation may range from 1% to 88%.^(6,8,9)^ However, prevalence data in this study suggest wider use of palliative sedation compared to other studies with physicians working in São Paulo,^(18,19)^ in which prevalence was 37% and 17%.^(18,19)^ Differences between studies may have been due to different prevalence assessment methods, different geographical locations and different numbers of participants. This is therefore thought to be the first study to assess the true prevalence of palliative sedation use by medical professionals working in São Paulo.

Results of this study indicate that, in spite of relatively high prevalence of palliative sedation use, frequency of use is low, since most physicians used the technique up to six times during the year prior to data collection. These findings are supported by literature data revealing high numbers of patients seen by hospital teams or in palliative care units,^(6,8)^ but few patients requiring palliative sedation over the course of in-hospital stay or treatment,^(8)^ and a prevailing frequency of use of up to five times.^(6,9)^ Nonetheless, direct comparisons regarding palliative sedation use prevalence and frequency data between this, Chater et al.,^(6)^ and Lux et al.,^(9)^ studies must be made with caution, since the latter studies involved palliative care specialists,^(6,9)^ while this sample comprised medical professionals of several specialties, some of whom may not deal directly with terminal diseases.

Pain was the major cause for palliative sedation implementation in this sample (35%). Pain complaint by terminal patients or by those not truly amenable to cure appears to be a significant factor in palliative care, given pain was also the major driver of palliative sedation use in eligible patients in previous studies.^(6,8,19)^ In contrast, lack of eligible patients was the major reason reported for not using palliative sedation over the last 12 months or for never having used it.

Studies have shown that decision about palliative sedation must be made together including the patient, family and healthcare team, or in an individualized manner.^(8,19)^ In this study, 95% of interviewees reported consulting or having consulted patient families prior to starting the procedure, and 86% believed family members may influence in establishing the practice at healthcare settings. Likewise, according to 43% of interviewees in the study of Spineli et al., the opinion of family member may be the major hurdle for palliative sedation implementation.^(19)^ Therefore, family decisions may interfere with palliative sedation practice and the relationship between families and multidisciplinary teams is vital to prevent suffering to those involved.^(19)^

As regards the level of comfort about drug use in palliative sedation, 83% of physicians reported feeling comfortable or very comfortable. However, only 26% of them sample had specific training in palliative care. These findings suggest poor technical background for provision of professional services in specific cases requiring palliative sedation, and may elicit related ethical debate, given specific academic training is a fundamental competence for qualified practice. According to Fonseca et al., lack of understanding may translate into malpractice, since the level of confidence of physicians is associated with their level of knowledge.^(16)^ Low number of physicians with specific training in palliative care may be justified by the fact that palliative care was not a formal specialty till recently,^(25,26)^ and has just been adopted by the National Health System (SUS).^(17)^ The relevance of established protocols at organizations must be emphasized in order to prevent medical error,^(14)^ indiscriminate use and higher health care costs.

In spite of potential contributions to increased understanding of palliative sedation in Brazil,^(17,27)^ limitations of this study must be accounted for when interpreting and extrapolating findings. The sample was limited to the State of São Paulo, where medical services are more abundant and palliative care-related research may be more advanced compared to other areas of the country. Furthermore, low adherence to the questionnaire (324; 2%), in contrast with 69% adherence reported in previous questionnaire-based studies investigating palliative care,^(27)^ may have failed to provide a representative sample of the São Paulo medical community. The fact that the questionnaire may have been completed solely by professionals inclined to contribute to palliative care research must be emphasized. Lack of incentives,^(28)^ limited awareness of the topic^(29)^ and non-anonymous online survey model may have contributed to low response rates.

## CONCLUSION

The prevalence of palliative sedation use in this sample was high, primarily for pain relief. In contrast, frequency of palliative sedation use was low, particularly due to lack of eligible patients. Most participants practice in different medical fields and had no specific training in palliative care. Palliative care is nonetheless an emerging practice and further studies are needed before it can be fully understood.

## Annex 1

**Table:** Palliative sedation questionnaire

Demographics
1) Gender:
( ) Female
( ) Male
2) Age:
( ) <30
( ) 30-39
( ) 40-49
( ) 50-59
( ) 60-69
( ) >70
3) State in which you currently practice:
( ) São Paulo
( ) Other
4) Medical specialty (more than one specialty accepted, ordered according to workload).
1.
2.
3.
5) Years in medical practice:
( ) 1 year
( ) 2 to 5
( ) 6 to 10
( ) 11 to 16
( ) >16 years
Palliative care
Definition: “Palliative care consists of assistance provided by a multidisciplinary team, aiming to improve the quality of life of patients and their families facing problems associated with life-threatening illness, through the prevention and relief of suffering, by means of early identification and impeccable assessment and treatment of pain and other physical, psychological, and spiritual symptoms” (Sepúlveda et al., 2002^(21)^).
6) How would you describe your practical experience with patients receiving palliative care at hospital?
( ) No practical experience
( ) Limited practical experience
( ) Moderate practical experience
( ) Wide practical experience
7) Are you trained in palliative care (undergraduate course, graduate course, other courses)?
( ) Yes
( ) No
Palliative sedation
Definition: “Deliberate administration of drugs that reduce the level of consciousness, with consent given by patient or guardian/person responsible, in order to properly relieve one or more refractory symptoms, in patients with advanced end-stage disease disease” (Kira, 2012;^(4)^ Ventafridda, 1990^(5)^).”
8) Are you in favor of palliative sedation as a therapeutic alternative in clinical practice?
( ) No
( ) Yes
9) Have you ever used palliative sedation as a therapeutic alternative?
( ) No*
( ) Yes**
* If no, go to question 10:
**If yes, go to question 11:
10) Why have you not used palliative sedation? Tick all applicable alternatives.
( ) Lack of eligible patients
( ) Not familiar with the procedure
( ) Believe it offends ethical principles
( ) Do not believe in treatment efficacy
( ) Other
Note: After question 10, go to question 19.
11) How would you describe your practical experience with palliative sedation?
( ) No practical experience
( ) Limited practical experience
( ) Moderate practical experience
( ) Wide practical experience
12) Have you practiced palliative sedation in the last 12 months?
( ) Yes*
( ) No**
* If yes, go to question 13:
** If no, go to question 18:
13) Why have you practiced palliative sedation in the last 12 months? Tick all applicable alternatives.
( ) Availability of eligible patients
( ) Treatment efficacy
( ) Only feasible treatment
( ) To alleviate pain
( ) Others
14) How many times have you practiced palliative sedation in the last 12 months?
( ) 1-6
( ) 7-12
( ) 13-18
( ) 19-24
( ) >24
15) What is your level of comfort with drug selection when using palliative sedation?
( ) Very comfortable
( ) Fairly comfortable
( ) Comfortable
( ) Not comfortable
( ) Not comfortable at all
16) Do you consult the family prior to initiating palliate sedation?
( ) Yes
( ) No
17) Do you believe family members influence in establishing palliative sedation in healthcare settings?
( ) Yes
( ) No
Note: After this question, go to question 19.
18) Why have you not practiced palliative sedation in the last 12 months? Mark all applicable alternatives.
( ) Lack of eligible patients for the procedure
( ) Treatment inefficacy
( ) It was not the only feasible treatment
( ) Would not alleviate pain
( ) Others
Note: After this question, go to question 19.
19) Do you believe palliative sedation accelerate end of life of patients progressing to death?
( ) Yes
( ) No
20) Do you believe physical symptoms influence emotional/psychosocial symptoms?
( ) Yes
( ) No
21) Would you use/have you used palliative sedation in patients with psycho-existential suffering? (hoplessness, dependence and lack of self-care ability, fear, death anxiety and panic, desire to control the moment of death, isolation and lack of social support).
( ) Yes
( ) No
22) Do you believe palliative sedation to be equivalent to euthanasia?
( ) Yes
( ) No
23) Is palliative sedation a well-established practice in your workplace?
( ) Yes
( ) No
