# Supplementary material for: Prevalence of palliative sedation in the State of São Paulo: an emerging medical demand
Source: Einstein (Sao Paulo). 2020 Sep 9;18:eAO5395. doi: 10.31744/einstein_journal/2020AO5395 (PMC7480494; doi:10.31744/einstein_journal/2020AO5395)
Supplement: Translation

# Translation: Prevalência de sedação paliativa no Estado de São Paulo: uma demanda médica emergente

**Authors:** Márjorie Anção Oliveira Piedade, Carlos Alberto Cardoso, Denise Gonçalves Priolli

**Affiliations:** 1 Brasil Universidade São Francisco, Bragança Paulista, SP, Brasil.; 2 Brasil Escola de Educação Física e Esporte, Universidade de São Paulo, São Paulo, SP, Brasil.

**Keywords:** Sedação profunda, Cuidados paliativos, Manejo da dor, Bioética, Assistência terminal, Planejamento antecipado de cuidados

## Abstract

**Objetivo:**

Verificar a prevalência e os fatores associados ao uso de sedação paliativa.

**Métodos:**

Trata-se de estudo observacional realizado a partir do preenchimento de um questionário eletrônico com 23 questões objetivas enviado a médicos paulistas. Foram avaliados os dados demográficos, a prevalência e a frequência de uso da sedação paliativa; o nível de conhecimento a respeito da técnica; e as motivações para sua utilização. Visando minimizar o viés de recordação, questões sobre a frequência de uso e motivações para tal, as respostas deveriam se referir ao último ano em relação à data de preenchimento da pesquisa. Foi utilizada estatística descritiva para sumarizar os resultados.

**Resultados:**

Foram enviados 20.168 e-mails e obtiveram-se 324 respostas válidas, resultando em 2% de adesão. A prevalência de utilização da sedação paliativa ao longo do histórico de atuação profissional foi de 68%, mas apenas 48% utilizaram a sedação paliativa no último ano, motivados, principalmente, pela presença de dor no paciente (35%). A frequência de uso foi de uma a seis vezes (66%), e a principal razão para não empregar a sedação paliativa foi a ausência de pacientes elegíveis (64%). Aproximadamente 83% dos médicos sentiam-se confortáveis quanto ao uso de sedação paliativa, mas apenas 26% possuíam formação teórica específica na área.

**Conclusão:**

A prevalência de uso da sedação paliativa é alta, sendo utilizada, na maioria das vezes, para proporcionar o alívio da dor do paciente. Por outro lado, sua frequência de utilização é baixa, principalmente pela ausência de pacientes elegíveis.

## INTRODUÇÃO

A manutenção da qualidade de vida do paciente com doença avançada e sintomas refratários^(1)^ é um grande desafio para os profissionais da saúde.^(2,3)^ Diante desse cenário, emergem estratégias, como a sedação paliativa, uma das técnicas dos cuidados paliativos que consiste na administração deliberada de fármacos a fim de reduzir o nível de consciência destes indivíduos e aliviar adequadamente um ou mais sintomas refratários.^(4,5)^

A sedação paliativa é utilizada, principalmente, para *delirium* hiperativo, dispneia, dor intensa intratável, sangramento e mioclonia incontroláveis,^(6,7)^ sendo seu emprego consentido pelo paciente ou seu responsável. Dados da literatura mostram que a prevalência de utilização da sedação paliativa é de 1% a 88% entre os profissionais da saúde.^(8,9)^ Esse amplo resultado poderia ser justificado por diferentes definições de sedação paliativa utilizadas nos estudos; diretrizes e culturas dos países em que a sedação paliativa é praticada;^(10-12)^ entendimento dos sintomas refratários; experiência e o conforto dos profissionais de saúde;^(9)^ local de aplicação da sedação paliativa; formas de avaliação da prevalência; e tipo de público entrevistado.^(8)^

Apesar de a sedação paliativa ser largamente utilizada para o tratamento dos sintomas físicos dos pacientes,^(6,7)^ ainda existem inconsistências na literatura quanto ao seu emprego para os sintomas psicoexistenciais,^(8,13)^ dada a dificuldade em diferenciar as respostas adequadas à doença e as psicopatologias, como a depressão. Além disso, tal técnica ainda representa um dilema terapêutico desafiador diante da eventual confusão existente entre sedação contínua profunda e eutanásia.^(8,14)^ A sedação paliativa contínua profunda suprime a consciência da pessoa doente até a morte, e a intenção é de aliviar o sofrimento. Já a eutanásia é definida como o ato de acelerar a morte do paciente, por meio de drogas em doses letais, e tem a intenção deliberada de abreviar a vida do paciente para interromper o sofrimento.^(14,15)^

No Brasil, as discussões sobre essa técnica são recentes,^(16)^ e não existem diretrizes específicas que abordem a indicação de sedação paliativa. Em 2009, o Conselho Federal de Medicina incluiu, no Código de Ética Médica, os cuidados paliativos como princípios fundamentais e, em 2011, formalizou a Medicina Paliativa como especialidade. Recentemente, foi publicada a resolução sobre a organização dos cuidados paliativos, no âmbito de Sistema Único de Saúde (SUS),^(17)^ no entanto, ainda não foi observado impacto significativo desta portaria nas práticas médicas.

Da mesma forma, pesquisas científicas relativas aos cuidados paliativos e à sedação paliativa praticadas por profissionais brasileiros são escassas. Sugere-se que a prevalência de sedação paliativa varie entre 17% e 37%,^(18,19)^ e as principais causas para sua utilização sejam a presença da dispneia, *delirium* e dor nos pacientes.^(19)^ Ressalta-se que a principal barreira descrita para a implementação de sedação paliativa é a influência da família.^(19)^ Tais pesquisas, porém, foram realizadas com um pequeno número amostral e centraram-se em pacientes ou equipes multidisciplinares de centros especializados, carecendo, ainda, de informações exclusivas a respeito da atuação médica frente a esta prática.

## OBJETIVO

Verificar a prevalência e a frequência de utilização da sedação paliativa no Estado de São Paulo, Brasil, as principais motivações e barreiras encontradas para a implementação da técnica, e se os médicos paulistas possuem formação específica em cuidados paliativos.

## MÉTODOS

### Desenho experimental

Foram utilizadas as recomendações *Strengthening the Reporting of Observational Studies in Epidemiology* (STROBE) para a redação deste artigo.^(20)^ Trata-se de pesquisa epidemiológica observacional e transversal, realizada a partir de um questionário eletrônico adaptado de dois estudos anteriores,^(9,19)^ e implementada pela plataforma Google Formulários. O questionário foi composto por 23 perguntas objetivas e distribuído para uma comunidade de médicos paulistas, entre março e dezembro de 2017 (Anexo 1).

### Participantes

Participaram desta pesquisa médicos que trabalhavam e moravam no Estado de São Paulo. O único critério de inclusão para a pesquisa foi ser médico com pelo menos 1 ano de experiência na prática clínica. Os voluntários foram excluídos quando não completaram a pesquisa ou duplicaram as respostas. Antes de responder à pesquisa, na primeira página do formulário, os participantes foram informados dos detalhes do estudo e preencheram o Termo de Consentimento Livre e Esclarecido.

### Procedimentos

Os participantes foram recrutados por meio de registros do Conselho Regional de Medicina do Estado de São Paulo (CREMESP). Foram realizadas três tentativas de envio para cada um dos 20.168 e-mails obtidos, com intervalo de 15 dias entre cada tentativa. Além disso, foi feita tentativa de contato com 32 sociedades médicas, 2 associações e 2 academias, solicitando que essas instituições encaminhassem o *link* do questionário para seus membros, ou disponibilizassem o acesso à pesquisa em seus sites oficiais.

### Questionário

Os participantes responderam ao questionário com 23 questões objetivas em plataforma online. Inicialmente foram questionados quanto aos dados demográficos (sexo, idade, tempo de atuação e especialidade). Na sequência, os participantes foram apresentados aos conceitos de cuidados paliativos e sedação paliativa.^(4,5,21)^ Todos os participantes foram perguntados se eram a favor da prática de sedação paliativa; se consideravam essa técnica equiparada à eutanásia e abreviação da vida; se a sedação paliativa estava bem institucionalizada no local de atuação profissional; se os sintomas físicos dos pacientes poderiam influenciar nos sintomas psicoexistenciais; e se fariam indicação de sedação paliativa para tratamento de sintomas não físicos.

Questionou-se também a prevalência de utilização de sedação paliativa. Para minimizar o viés de recordação, determinadas questões foram restritas ao período de 12 meses que antecederam a data de preenchimento do questionário,^(22)^ tais como a prevalência de uso neste período, os principais motivadores, a frequência de utilização de sedação paliativa, o nível de conforto em relação à administração de fármacos, a formação específica em cuidados paliativos e a influência da família do paciente na decisão de utilizar sedação paliativa. Aos médicos que nunca empregaram sedação paliativa ou não usaram essa técnica no último ano em relação a data de preenchimento do questionário, foi perguntado o principal motivo para tal ação.

### Análise estatística

Os resultados foram sumarizados por meio da estatística descritiva e análises de frequência. Todas as análises foram conduzidas no IBM^®^ SPSS^®^ Statistics versão 23.

A pesquisa foi realizada de acordo com as Normas e Diretrizes Éticas, tendo sido aprovada pelo Comitê de Ética em Pesquisa da Universidade São Francisco (número 1.964.009, CAAE: 65240217.4.0000.5514).

## RESULTADOS

Dos 20.168 e-mails enviados e dos contatos realizados com as instituições, obtiveram-se 338 questionários respondidos. Foram excluídos 14 questionários, 12 apresentaram duplicidade, e 2 estavam com campos incompletos, resultando em 324 questionários selecionados para análise.

A caracterização demográfica dos voluntários encontra-se disponível na tabela 1. Dentre os participantes, 51% eram do sexo masculino (n=165). A idade prevalente da amostra se encontrava entre 30 a 39 anos (34%), e 44% possuía mais de 16 anos de atuação profissional. Trezentos e catorze entrevistados eram especialistas em alguma área médica (97%) e, dentre as áreas médicas reportadas, houve predomínio da clínica médica (n=167).

**Tabela 1:** Características socioprofissionais dos médicos com 1 ano ou mais de atuação

Sexo	
Masculino	165 (51)
Feminino	159 (49)
Idade, anos	
<30	55 (17)
30-39	110 (34)
40-49	6 (20)
50-59	47 (19)
60-69	42 (13)
>70	7 (1)
Tempo de atuação, anos	
1	19 (6)
2-5	7 (22)
6-10	4 (15)
11-16	45 (14)
>16	141 (44)
Especialista em área médica	
Sim	314 (97)
Não	10 (3)
Especialidades	
Clínica	167 (53)
Cirúrgica	60 (19)
Pediatria	26 (8)
Psiquiatria	17 (5)
Ginecologia e Obstetrícia	16 (5)
Medicina da Família	10 (3)
Medicina do Trabalho	8 (3)
Anestesiologia	6 (2)
Outros	4 (1)

Resultados expressos em n (%).

Dentre os entrevistados, 99% relataram que eram favoráveis à prática de sedação paliativa. A maioria dos médicos não acreditava que a sedação paliativa fosse equiparada com a eutanásia (312; 99%) ou que abreviasse a vida do paciente (278; 83%). Porém, cerca de 60% acreditavam que a sedação paliativa não estivesse bem institucionalizada no local onde atuavam (n=195). Todos os médicos acreditavam que os sintomas físicos influenciam em sintomas psicoexistenciais, mas 61% relataram que não indicariam a sedação paliativa para os pacientes com tais sintomas não físicos (n=198).

Quando perguntados sobre a utilização da sedação paliativa, 68% relataram já terem empregado essa técnica como opção de tratamento em pacientes elegíveis (n=221). Considerando apenas o período dos últimos 12 meses em relação à data de preenchimento do questionário, a prevalência de utilização da sedação paliativa foi de 48% (n=155). A principal causa reportada para a utilização da sedação paliativa foi proporcionar o alívio da dor do paciente (n=97; 35%) (Figura 1), e a 66% dos entrevistados relataram ter utilizado a sedação paliativa entre uma e seis vezes durante o ano (n=103) (Figura 2). Cerca de 83% dos profissionais que a usaram nos últimos 12 meses, sentiam-se confortáveis ou muito confortáveis com a seleção de drogas (n=129) (Figura 3). No entanto, apenas 26% dos profissionais que já utilizaram sedação paliativa relataram possuir formação específica em cuidados paliativos (n=41). Além disso, 95% dos médicos consultavam ou tinham consultado a família do paciente antes da realização do procedimento (n=148), e 86% acreditavam que esta podia influenciar na institucionalização da prática (n=133).

**Figura 1:** Distribuição de médicos com 1 ano ou mais de atuação ao responderem sobre as causas para a prática de sedação paliativa

**Figura 2:** Distribuição de médicos com 1 ano ou mais de atuação ao responderem sobre a frequência de utilização de sedação paliativa

**Figura 3:** Distribuição de médicos com 1 ano ou mais de atuação ao responderem sobre o nível de conforto em relação à seleção de drogas na prática em sedação paliativa

A principal razão reportada para a não utilização de sedação paliativa dentre aqueles que nunca usaram a técnica ou que não a usaram no período dos últimos 12 meses foi a ausência de pacientes elegíveis − 64% (n=66) e 100% (n=66), respectivamente. O desconhecimento do procedimento (n=35; 34%) e outras razões não especificadas (n=2; 2%) foram outras justificativas listadas para nunca terem utilizado esta técnica.

## DISCUSSÃO

O principal achado deste estudo foi o de que a prevalência de utilização da sedação paliativa entre os médicos paulistas é alta. Porém, quando o período de análise foi restrito ao último ano, a prevalência caiu, e a frequência de uso foi relativamente baixa. Observou-se, também, que apenas uma minoria dos médicos possuía formação específica em cuidados paliativos, e a principal razão para o emprego da sedação paliativa era a presença de dor no paciente.

Nossa amostra foi caracterizada predominantemente por pessoas do sexo masculino (51%), por profissionais com mais de 16 anos de atuação, e com idade entre 30 e 39 anos. Além disso, 97% da amostra é especialista em alguma área da medicina. Tais resultados se assemelham aos dados disponibilizados pelo CREMESP, em 2018, segundo os quais 55% dos 126.687 médicos em atividade no Estado de São Paulo eram homens, com média de idade de 45 anos, e com 20,2 anos de tempo de formado, além de 66% possuírem especialidade em alguma área médica.

Apesar de a literatura sugerir que existem limites tênues na distinção entre sedação paliativa e eutanásia, e que pode existir ainda uma confusão no entendimento das duas práticas,^(14,15)^ os resultados deste estudo indicaram que os profissionais da saúde participantes conseguem perceber diferenças entre ambas, fato este que poderia legitimar o uso da sedação paliativa em pacientes elegíveis. Resultados semelhantes foram observados anteriormente,^(15,19)^ em que os profissionais da saúde que trabalham em um setor hospitalar de cuidados paliativos oncológicos,^(15)^ bem como os profissionais que trabalham ou trabalharam em centros especializados de cuidados paliativos,^(19)^ conseguem ter clara distinção entre os termos, tendo a ética como alicerce fundamental para o exercício da sedação paliativa.

Ainda que todos os entrevistados acreditem que os sintomas físicos influenciem nos sintomas emocionais, nossos resultados indicaram que a maioria dos profissionais não utilizaria a sedação paliativa para tratar pacientes com dor psicoexistencial. Enquanto alguns autores encontraram aumento na indicação de sedação paliativa para tratar sintomas psicoexistenciais, justificado pelos princípios bioéticos.^(23)^ Outros autores, porém, contraindicam a sedação paliativa para sintomas não físicos, pois afirmam que nem sempre significa eminência de morte.^(24)^

A prevalência de sedação paliativa encontrada nesse estudo foi de 68%, quando considerado todo o histórico de atuação profissional dos voluntários, e de 48%, quando o período de utilização esteve restrito ao último ano. Tais resultados se encontram dentro do sugerido pela literatura, de que a prevalência de sedação paliativa pode variar entre 1% a 88%.^(6,8,9)^ Por outro lado, nossos resultados sugerem maior uso da sedação paliativa em comparação aos demais estudos com profissionais paulistas,^(18,19)^ que apontaram prevalência de 37% e 17%.^(18,19)^ As diferenças entre os resultados poderiam ser justificadas pelos métodos utilizados em cada estudo para mensurar a prevalência de uso e pelos locais de realização, além do número de participantes. Desta forma, pelo melhor do nosso conhecimento, trata-se do primeiro estudo a acessar a real prevalência de utilização da sedação paliativa por profissionais médicos paulistas.

Nossos resultados indicaram que, apesar da prevalência de uso da sedação paliativa ser relativamente alta, sua utilização é pouco frequente, visto que a maioria dos médicos utilizou esta técnica por até seis vezes ao longo do último ano. A literatura corrobora esses achados, pois mostrou que o número de pacientes atendidos por equipes em hospitais ou em unidades de cuidados paliativos é alto,^(6,8)^ mas poucos pacientes podem necessitar do emprego da sedação paliativa durante o período de internação ou de tratamento,^(8)^ indicando que a frequência de uso da técnica, predominantemente, foi de até cinco vezes.^(6,9)^ Por outro lado, as comparações diretas a respeito da prevalência e da frequência de uso da sedação paliativa entre nossos achados e os resultados de Chater et al.,^(6)^ e de Lux et al.,^(9)^ devem ser feitas com cautela. Ambos avaliaram especialistas na área dos cuidados paliativos,^(6,9)^ e nossa amostra foi composta por profissionais médicos de diversas especialidades, de modo que alguns dos participantes podem não atuar diretamente com doenças que possam causar terminalidade.

A principal causa relatada para a implementação da sedação paliativa foi a dor do paciente (35%). A presença de dor no doente em fase terminal ou fora de possibilidades reais de cura parece ser um importante elemento para ser considerado durante os cuidados paliativos, visto que, a dor também foi o principal motivador para a utilização da sedação paliativa nos pacientes elegíveis em estudos anteriores.^(6,8,19)^ Em contrapartida, a ausência de pacientes elegíveis foi o principal fator reportado para a não aplicação de sedação paliativa nos últimos 12 meses ou para nunca ter empregado essa técnica.

Estudos mostram que a decisão pela sedação paliativa deve ser tomada em conjunto entre paciente, família e equipe de saúde, ou de forma individualizada.^(8,19)^ Dentre os entrevistados neste estudo, 95% consultam ou consultaram a família do paciente antes da realização do procedimento, e 86% acreditam que ela pode influenciar na institucionalização. Da mesma forma, para 43% dos entrevistados no estudo de Spineli et al., a opinião dos familiares pode ser a principal barreira para instituir a sedação paliativa,^(19)^ de modo que, a decisão da família do paciente pode interferir na prática de sedação paliativa, e sua relação com a equipe multidisciplinar é imprescindível para evitar o sofrimento dos envolvidos.^(19)^

Quanto ao nível de conforto em relação à utilização de drogas na prática de sedação paliativa, 83% dos médicos relataram se sentirem confortáveis ou muito confortáveis. Por outro lado, apenas 26% dos médicos de nosso estudo possuíam formação teórica específica em cuidados paliativos. Estes resultados sugerem baixa preparação técnica para a atuação profissional em casos específicos que necessitem de sedação paliativa e podem trazer questionamentos éticos quanto essa prática, já que a formação específica serve como competência essencial para realização deste exercício qualificado. Fonseca et al., destacam que a falta de conhecimento pode alicerçar a má conduta, já que a segurança do médico está relacionada ao nível de conhecimento que possui.^(16)^ A baixa quantidade de médicos com formação específica no tema neste estudo poderia ser justificada pela ausência de formalização quanto aos cuidados paliativos,^(25,26)^ somada às recentes incorporações destas técnicas no SUS.^(17)^ Desta forma, ressalta-se a importância de protocolos institucionalizados, evitando a possibilidade de erro médico,^(14)^ sua utilização sem critério e maiores custos ao sistema de saúde.

Embora existam esforços para ampliar o entendimento da sedação paliativa no Brasil,^(17,27)^ o presente estudo apresenta algumas limitações, que devem ser consideradas no momento de interpretar e extrapolar estes resultados. A amostra foi restrita ao estado de São Paulo, cuja atuação médica é mais concentrada, além de ser um local onde os estudos a respeito dos cuidados paliativos podem estar mais avançados em relação ao restante do país. Além disso, a baixa adesão ao questionário (324; 2%), contrapondo-se à adesão de 69% observada previamente em estudo que utilizou questionário para tratar do tema de cuidados paliativos,^(27)^ não resulta, necessariamente, em uma amostra representativa da população médica atuante no estado de São Paulo. Destaca-se ainda que o questionário pode ter sido respondido somente por profissionais que estejam interessados em contribuir com os avanços das pesquisas científicas na área dos cuidados paliativos. Ademais, a ausência de incentivos,^(28)^ a pouca sensibilização sobre o assunto,^(29)^ ou o fato de ter sido uma pesquisa online, com identificação do participante, podem ter levado a uma diminuição na taxa de respostas.

## CONCLUSÃO

A prevalência de utilização de sedação paliativa na amostra avaliada foi elevada, sendo empregada, na maioria das vezes, em situações de dor no paciente. Por outro lado, sua frequência de utilização foi baixa, principalmente pela ausência de pacientes elegíveis. Apesar da maioria dos participantes apresentarem atuações médicas distintas e não possuírem formação específica em cuidados paliativos, trata-se de uma prática emergente, e mais estudos ainda se fazem necessários para seu completo entendimento.

## Anexo 1

**Table:** Questionário sedação paliativa

Estudo demográfico
1) Gênero:
( ) Feminino
( ) Masculino
2) Idade:
( ) <30
( ) 30-39
( ) 40-49
( ) 50-59
( ) 60-69
( ) >70
3) Em qual estado você atua no momento?
( ) São Paulo
( ) Outros
4) Especialidade médica (pode ser utilizada mais que uma especialidade, coloque na ordem de carga horaria).
1.
2.
3.
5) Quantos anos atua na área médica?
( ) 1 ano
( ) 2 a 5
( ) 6 a 10
( ) 11 a 16
( ) >16 anos
Cuidados paliativos
Definição: “Cuidados paliativos consistem na assistência promovida por uma equipe multidisciplinar, que objetiva a melhoria da qualidade de vida do paciente e seus familiares, diante de uma doença que ameace a vida, por meio da prevenção e alívio do sofrimento, da identificação precoce, avaliação impecável e tratamento de dor e demais sintomas físicos, sociais, psicológicos e espirituais” (Sepúlveda et al., 2002^(21)^).
6) Qual é a sua experiência prática com pacientes que recebem cuidados paliativos no hospital?
( ) Nenhuma experiência prática
( ) Pouca experiência prática
( ) Experiência prática moderada
( ) Muita experiência prática
7) Você possui formação em cuidados paliativos (graduação, pós graduação, cursos)?
( ) Sim
( ) Não
Sedação paliativa
Definição: “É a administração deliberada de fármacos que reduzem o nível de consciência, com o consentimento do paciente ou de seu responsável, que tem como objetivo aliviar adequadamente um ou mais sintomas refratários em pacientes com doença avançada terminal” (Kira, 2012;^(4)^ Ventafridda et al., 1990^(5)^).
8) Você é a favor da prática clínica de sedação paliativa como opção terapêutica?
( ) Não
( ) Sim
9) Você já utilizou sedação paliativa como opção de tratamento?
( ) Não*
( ) Sim**
* Em caso negativo, é direcionado para a pergunta 10:
**Em caso afirmativo, é direcionado para a pergunta 11:
10) Por que você não utilizou sedação paliativa? Marque todas que se apliquem.
( ) Não havia pacientes elegíveis
( ) Não conhece o procedimento
( ) Acredita que fere os princípios éticos
( ) Não acredita na eficácia do tratamento
( ) Outro
Obs: Após esta pergunta, vá para a pergunta 19.
11) Qual a sua experiência prática no uso de sedação paliativa?
( ) Nenhuma experiência prática
( ) Pouca experiência prática
( ) Experiência prática moderada
( ) Muita experiência prática
12) Você implementou a sedação paliativa nos últimos 12 meses?
( ) Sim*
( ) Não**
* Em caso afirmativo, vá para a pergunta 13:
**Em caso negativo, vá para a pergunta 18:
13) Porque você implementou a sedação paliativa nos últimos 12 meses? Marque todas que se apliquem.
( ) Havia pacientes elegíveis para o procedimento
( ) Eficácia do tratamento
( ) Único tratamento viável
( ) Proporcionar o alívio da dor
( ) Outros
14) Quantas vezes nos últimos 12 meses, você implementou a sedação paliativa?
( ) 1-6
( ) 7-12
( ) 13-18
( ) 19-24
( ) >24
15) O quão confortável você se sente com a seleção das drogas quando utiliza a sedação paliativa?
( ) Muito confortável
( ) Um pouco confortável
( ) Confortável
( ) Nem um pouco confortável
( ) Nada confortável
16) Você consulta a família antes de instituir a sedação paliativa?
( ) Sim
( ) Não
17) Você acredita que a família influencia na institucionalização da sedação paliativa?
( ) Sim
( ) Não
Obs: Após esta pergunta, vá para a pergunta 19.
18) Porque você não implementou a sedação paliativa nos últimos 12 meses? Marque todas que se apliquem.
( ) Não havia pacientes elegíveis para o procedimento
( ) Ineficácia do tratamento
( ) Não era o único tratamento viável
( ) Não iria proporcionar o alívio da dor
( ) Outros
Obs: Após esta pergunta, vá para a pergunta 19.
19) Você acredita que o uso de sedação paliativa abrevia a vida do paciente em processo de morte?
( ) Sim
( ) Não
20) Você acredita que os sintomas físicos influenciam nos sintomas emocionais/psicossociais?
( ) Sim
( ) Não
21) Você faria/fez uso da sedação paliativa em pacientes com sofrimento psicoexistencial? (desesperança, dependência e inabilidade para o autocuidado, medo, ansiedade e pânico da morte, desejo de controlar o momento da morte, isolamento e ausência de suporte social).
( ) Sim
( ) Não
22) Você acredita que a sedação paliativa é equiparada a eutanásia?
( ) Sim
( ) Não
23) No seu meio médico atual, a sedação paliativa está bem institucionalizada?
( ) Sim
( ) Não
